# Evaluation of the Sustaining Effects of Tai Chi Qigong in the Sixth Month in Promoting Psychosocial Health in COPD Patients: A Single-Blind, Randomized Controlled Trial

**DOI:** 10.1155/2013/425082

**Published:** 2013-10-24

**Authors:** Aileen W. K. Chan, Albert Lee, Diana T. F. Lee, Janet W. H. Sit, S. Y. Chair

**Affiliations:** ^1^The Nethersole School of Nursing, The Chinese University of Hong Kong, Shatin, N.T., Hong Kong; ^2^The Jockey Club School of Public Health and Primary Care, The Chinese University of Hong Kong, Shatin, N.T., Hong Kong

## Abstract

*Objectives*. To evaluate the sustaining effects of Tai Chi Qigong (TCQ) in improving the psychosocial health in chronic obstructive pulmonary disease (COPD) patients in the sixth month. *Background*. COPD affects both physical and emotional aspects of life. Measures to minimize patients' suffering need to be implemented. *Methods*. 206 COPD patients were randomly assigned into three groups: TCQ group, exercise group, and control group. The TCQ group completed a three-month TCQ program, the exercise group practiced breathing and walking exercise, and the control group received usual care. *Results*. Significant group-by-time interactions in quality of life (QOL) using St. George's respiratory questionnaire (*P* = 0.002) and the perceived social support from friends using multidimensional scale of perceived social support (*P* = 0.04) were noted. Improvements were observed in the TCQ group only. *Conclusions*. TCQ has sustaining effects in improving psychosocial health; it is also a useful and appropriate exercise for COPD patients.

## 1. Introduction

Chronic obstructive pulmonary disease (COPD) ranks as the fourth leading cause of death worldwide [1]. In terms of morbidity, COPD ranks 13th. The World Health Organization estimates that the disease will become the third global killer by 2030. COPD is a progressive disease, and the lung functions of COPD patients may continue to deteriorate [2]. Manifestations of COPD include fatigue, weakness, activity intolerance, and dyspnoea. As the disease worsens, its signs and symptoms become more obvious, and the patient's ability to perform daily activities is affected [3]. Patients may become increasingly dependent on others, may stop participating in activities, and finally may isolate themselves [4]. The effect of the disease on both physical and emotional aspects of life may lead to disability and an impaired emotional state [5], which would in turn influence the quality of life. If COPD patients are better supported and cared for, these negative psychosocial consequences may be prevented or mitigated [6]. Therefore, measures to minimize patients' suffering need to be implemented [7]. With existing evidence supporting the benefits of exercise in COPD patients [8], Tai Chi Qigong may be an alternative exercise for this group of people. Tai Chi Qigong is a form of gentle exercise suitable for older adults and relatively frail people because it can be mastered without much exertion [9, 10]. These known characteristics of Tai Chi Qigong match with the background of COPD patients who may not have good physical statuses.

Qigong is an ancient Chinese system of gentle self-healing exercises that are designed to cultivate functional integrity and enhance the life vital energy called *Qi*. Tai chi is a martial art that can be viewed as a therapeutic exercise. Tai chi practice involves the recognition, development, and use of *Qi*. This vital energy flows in the body along channels called meridians and collaterals, which connect all the organ systems and tissues. When *Qi* is abundant, flowing freely, and in balance, a person usually enjoys good health and longevity. However, when *Qi* becomes deficient, excessive, stagnant, or blocked, diseases will occur. Tai chi and Qigong can be combined to promote good circulation of *Qi* in the body. These techniques can be learned and practiced by people with various problems to maintain general health and wellness. The health benefits of Tai Chi Qigong, particularly in COPD patients, have been rarely studied in healthcare research, so a randomized controlled trial was conducted to address this knowledge gap and to investigate the short-term therapeutic effect of Tai Chi Qigong in COPD patients [11]. After the three-month intervention period, the effect of practicing Tai Chi Qigong on health-related quality of life (HRQL) was observed in symptom and activity domains using the St George's respiratory questionnaire (SGRQ). Given that Tai Chi Qigong is a complex exercise routine that requires considerable practice to attain proficiency, whether its effects are sustained over a longer time frame must be investigated. Therefore, this study explored the sustaining effects of Tai Chi Qigong on HRQL and its longer-term effects on perceived social support in the sixth month from baseline with continuing regular self-practice of Tai Chi Qigong.

## 2. Methods

### 2.1. Study Design and Methods

This study used a single-blind, randomized controlled trial with repeated measures. Research assistants for data collection were blind to the study to minimize researcher bias.

### 2.2. Subjects and Study Setting

The subjects were recruited from five general outpatient clinics; informed consent was obtained prior to the study. Ethical approval and permissions for conducting the study in the selected general outpatient clinics were also obtained. Inclusion criteria for subject recruitment were the following: clinically diagnosed with COPD, as defined by the American Thoracic Society [12], and able to walk independently. Subjects were excluded if they had suffered from severe sensory or cognitive impairment, symptomatic ischemic heart disease, or had practiced Tai Chi Qigong within a year prior to the commencement of the study.

### 2.3. Sample Size and Randomization

The sample size was based on previous findings on the medium effect of Tai Chi Qigong exercises on HRQL in COPD patients [13]. With a power of 0.80, at a 5% significant level of repeated measure ANOVA, 52 subjects per group were required [14]. To cover for the potential attrition rate of 25% [15], a total of 206 subjects were recruited and randomly assigned to one of the three groups, namely, Tai Chi Qigong (TCQ) group (*n* = 70), exercise group (*n* = 69), and control group (*n* = 67). Random allocation was done by a computer-generated randomizer [16]. 

### 2.4. Intervention

Subjects in the TCQ group completed a Tai Chi Qigong program consisting of two 60-minute sessions each week for three months. They were advised to continue daily self-practice of Tai Chi Qigong upon completion of the instructor-led program. Subjects in the exercise group were taught to practice breathing exercise combined with walking as an exercise. They were also advised to self-practice the breathing and walking exercises daily. A diary was given to the subjects in both the TCQ and exercise groups to record the frequency of their self-practice.

Subjects in the control group were instructed to maintain their usual daily activities. No extra exercise was recommended. To enhance the internal validity of the study findings, participants in the exercise and control groups were designated to join nonexercise community activities in the three-month Tai Chi Qigong training period. The purpose of this arrangement was to maintain regular gatherings for all participants to balance the emotional effect of the extra weekly gatherings of the TCQ group during the process of Tai Chi Qigong training.

During the study period, all subjects continued their prescribed medical treatments. Data collections were performed at baseline (T1), sixth week (T2), third month (T3), and sixth month (T4). 

### 2.5. Outcome Measures

The outcome measures were HRQL using SGRQ, clinical significance of SGRQ using minimum clinically important difference (MCID), and self-perceived social support using multidimensional scale of social support (MSPSS).

SGRQ is a disease-specific measure of HRQL in COPD patients. Score ranges from 0 to 100, where 0 indicates “best health” and 100 indicates “worst health” [17]. SGRQ has been tested to be a valid, sensitive, and reliable instrument that can be used to assess HRQL in western and Chinese people with COPD [18, 19]. Cronbach's *α* ranges from 0.74 to 0.95. Test-retest reliability shows intraclass correlation coefficients of all the dimensions exceeding 0.70 (*P* < 0.001).

MCID is defined as the smallest difference in the client perceived as beneficial or important [20, 21]. It is used to evaluate the clinical significance of the effects of SGRQ perceived by patients. The threshold for each of the SGRQ domains is 4 units [22].

MSPSS is a 12-item questionnaire that examines the self-perceived social support from social relationships, including family, friends and significant others [23]. It uses a seven-point Likert scale set from 1 (strongly disagree) to 7 (strongly agree). The total score ranges from 12 to 84, with higher scores indicating higher levels of perceived support. The psychometric properties of the Chinese version demonstrate high internal consistency, with a Cronbach's *α* equal to 0.89; its validity and reliability have been confirmed [24].

### 2.6. Statistical Analysis

Data analyses were conducted using SPSS version 18.0. Descriptive statistics were used to describe the demographic characteristics of the sample. Repeated measure analysis of covariance (ANCOVA) was used to examine the outcome measures. A *P* value of 0.05 was used as the level of statistical significance. An intention-to-treat analysis was applied in calculating the missing values. In case of withdrawals, the data of the last observation were carried forward [25].

## 3. Results

**Figure 1:**
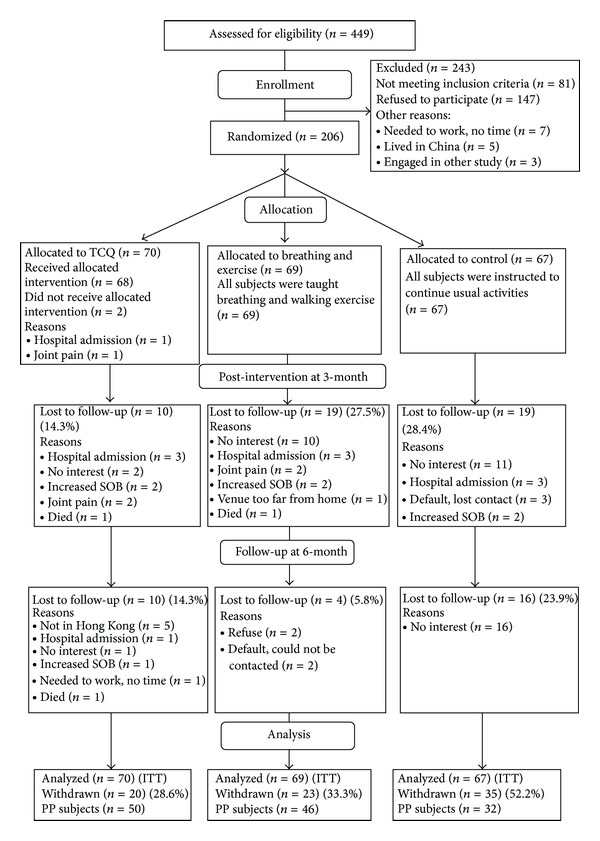
CONSORT (2005) flowchart to track participants through randomized controlled trail. ITT: intention to-treat; PP: per protocol [39].

A total of 206 subjects participated in this study, among which 158 completed the study program at the third month. The attrition rate was 23.8%. A total of 128 subjects (TCQ group = 50, exercise group = 46, and control group = 32) received follow-up assessment in the sixth month (Figure 1). No statistically significant differences were observed in the demographic data among the three study groups, except for gender (Table 1). This finding was ascribed to the lesser number of females (9.2%) who participated in this study. The obvious difference in the number of male and female participants may be because of the fact that COPD is more common in men [26]. Therefore, the confounding effect of gender was controlled as a covariate in the data analyses.

**Figure 2:**
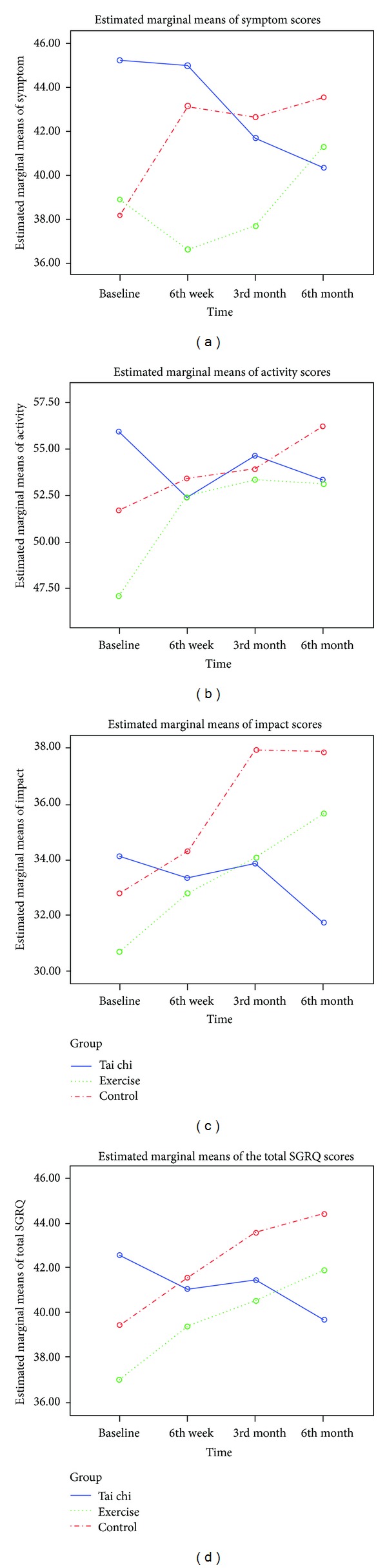
Mean scores of the four domains of St George's respiratory questionnaire (SGRQ) among the three study groups from baseline to 6th month.

Table 2 presents the descriptive statistics and the results of repeated measures ANCOVA of SGRQ. Improvements were shown in all domains of the SGRQ in the TCQ group, whereas deteriorations were noted both in the exercise group and in the control group. Significant group-by-time interaction effects revealed group differences across time, with the TCQ group having statistical improvement in symptom domain (*F* (6, 606) = 3.959, *P* < 0.001), activity domain (*F* (6, 606) = 2.418, *P* = 0.026), impact domain (*F* (6, 606) = 2.344, *P* = 0.030), and total SGRQ score (*F* (6, 606) = 3.510, *P* = 0.002) across the six-month study period compared with the exercise and control groups (Figure 2).

Improvements in self-perceived symptom score by 4.6 units, activity score by 2.5 units, impact score by 1.6 units, and total score by 2.4 units were observed in the TCQ group over the six-month study period. Deteriorations were noted in the exercise group in the symptom score by 2.3 units, activity score by 6.0 units, impact score by 4.6 units, and total score by 4.6 units from baseline to the sixth month. Worsening health status was found in all aspects in the control group. The declines were indicated by the increase of symptom score by 5.2 units, activity score by 4.5 units, impact score by 4.7 units, and total score by 4.7 units.

**Figure 3:**
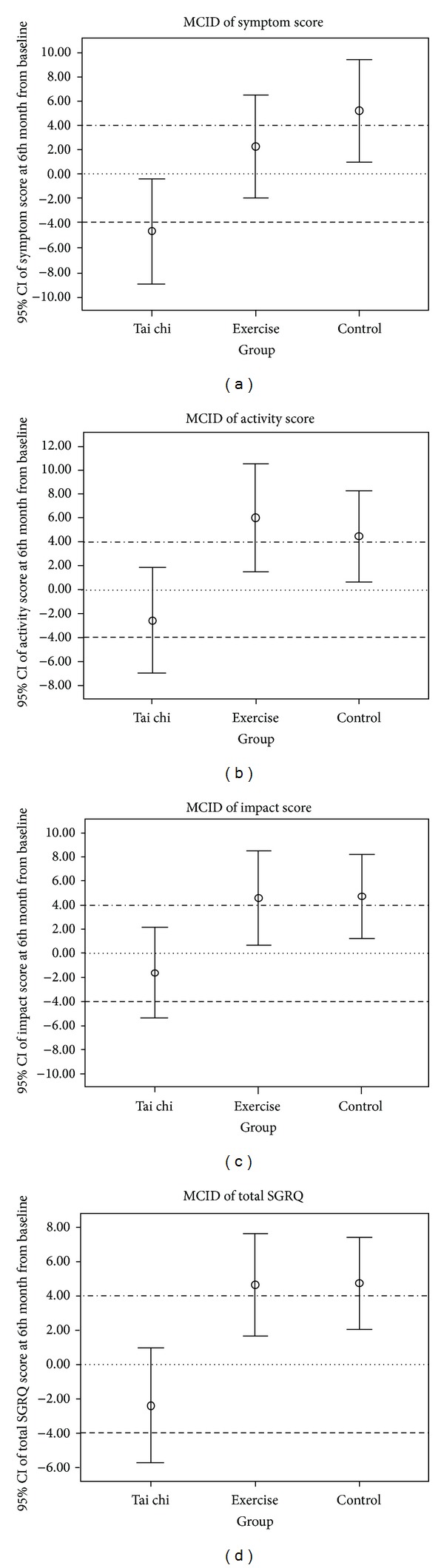
Minimum clinically important difference (MCID): changes in the St. George's respiratory questionnaire (SGRQ) scores at 6th month from baseline. ∘ Circles represent the mean; whiskers represent 95% confidence intervals; (dotted line) no change from baseline; (dashed line) (−4) threshold for positive clinical significance; (double-dotted line) (+4) threshold for negative clinical significance.

For SGRQ, a decrease of more than 4 units in the score is indicative of clinical significance with positive functional change [27]. The current study reported a statistically significant improvement in HRQL in the TCQ group. However, apart from the symptom domain, the statistically significant results were not clinically significant. By contrast, the exercise group demonstrated clinically significant deterioration in all findings, except in the symptom domain. Worsening health status was found in all aspects of the SGRQ, which was clinically significant in the control group (Figure 3).

**Figure 4:**
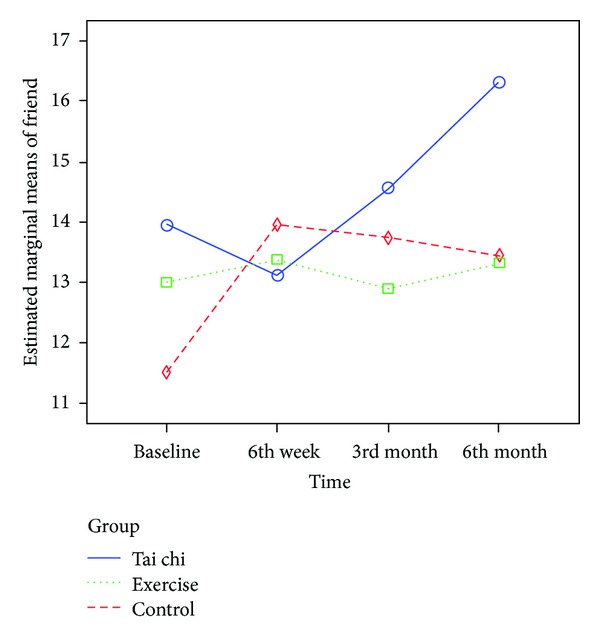
Mean scores of perceived social support from friends from baseline to 6th month.

Regarding the multidimensional scale of perceived social support, significant time-by-group interaction effect was noted in the perceived social support from friends (*F* (4.68, 472.47) = 2.338, *P* = 0.044), with improvement (+16.6%) found in the TCQ group (Figure 4). No differences were found in the perceived social support from family (*P* = 0.602) and significant others (*P* = 0.101) and in the total score (*P* = 0.056). Furthermore, no significant changes were found in perceived social support both in the exercise and control groups (Table 3, Figure 4).

## 4. Discussion

Psychological well-being is an important factor in rehabilitation for chronic illnesses. The effect of COPD on HRQL has been extensively explored in the United States and in Europe (*n* = 3265), with 61% of COPD patients stating that their health is fair to very poor [28]. This study adopted SGRQ to measure the HRQL of COPD patients. SGRQ is one of the most widely used disease-specific health status measures for COPD. It has been tested in a group of healthy subjects (*n* = 74), and the mean scores for the symptom, activity, impact, and total scores are 12, 9, 2, and 6, respectively [19]. In this study, the COPD participants reported a low level of HRQL at baseline using SGRQ, and the mean scores for the four captioned domains were 41, 52, 33, and 40, respectively, indicating that their HRQL was affected by the disease. These findings concur with the existing evidence that COPD affects HRQL [29].

Previous studies have shown that the beneficial effects on HRQL are gained during inpatient pulmonary rehabilitation; however, the effects decrease and cannot be sustained after discharge [29, 30]. The findings may be ascribed to the fact that patients revert to their prerehabilitation sedentary lifestyles after being discharged from hospitals. The other reason may be the severely impaired health status in COPD patients, which impedes their ability to achieve a higher response to rehabilitation programs. Patients with moderate to severe COPD are often unable to sustain high-intensity exercise. Therefore, the low-intensity Tai Chi Qigong exercise with 3.1 MET [31] was adopted as intervention for the COPD patients in this study. This intensity of Tai Chi Qigong exercise was well tolerated and enjoyed by the participants. The perceived positive effects elicited demands from the COPD patients to continue the Tai Chi Qigong exercise upon completion of the three-month training program. Majority of the participants (93%) continued practicing Tai Chi Qigong after they learned the skills. The positive effects were reflected on their adherence to regular Tai Chi Qigong practice and the significant improvements in their HRQL within three months on a low-intensity Tai Chi Qigong program conducted twice a week. The results were substantial after another three months of self-practice, suggesting that additional health gains could be derived from a longer period of practice. By contrast, subjects in the other two groups perceived a continued deteriorating trend in HRQL from baseline to the sixth month, which matched with the literature stating that COPD patients exhibit progressive decline in physiological and psychosocial functions [2, 32]. Tai Chi Qigong appeared to be more beneficial than the popular breathing and walking exercises often used as pulmonary rehabilitation intervention in COPD patients.

### 4.1. Social Support

Subjective perceived social support is important in improving health management and in adhering to the demanding treatment regimen of COPD patients [7, 33]. Thus, interventions should include efforts to strengthen social networks. No prior study has examined the effect of Tai Chi Qigong on social support among COPD patients. In the current study, the effectiveness of Tai Chi Qigong in promoting perceived social support in patients was investigated. MSPSS was adopted for the measurement of social support. Baseline findings showed that the participants perceived best social support from family members with the mean item score of 5.45, which fell well above the midpoint of 3.5. The mean item score of perceived social support from significant others was 3.74, which fell around the midpoint of 3.5, indicating that the participants perceived themselves to be fairly supported by their significant others. The perceived social support from friends was the lowest among the three categories, with the mean item score of 3.21, which fell below the midpoint of 3.5, suggesting that the participants perceived themselves having a limited social support network from friends. This finding concurred with previous local studies in the Chinese elderly, which reported that the social support for Chinese older adults mainly came from family members [34, 35]. The high average mean for family support in the current study is also congruent with other local studies by Jiang et al. [36] in Chinese COPD patients. This observation could be explained in the Chinese tradition that people tend to maintain strong and cohesive bond between family members, indicating they have a limited social network size.

Social support is a high-level functioning factor for improving psychosocial health. Previous studies have reported positive effects of group exercise on psychological health for clients with similar diseases [37]. Group intervention provides a psychological and socialization context for support, and this intervention matches with the TCQ group intervention in this study. Although individual exercise can result in psychological enhancement, group dynamics further improve such opportunities. Tai Chi Qigong would help subjects improve their concept of self, which would increase their intention to adopt a particular course of health- or illness-related action [38].

During the first three months of the Tai Chi Qigong training period, arrangements were made for all subjects in the current study to attend regular weekly gatherings. Although the outcome of perceived social support was insignificant during the first three months, it became substantial on the sixth month follow-up assessment. This finding may be due to the majority (93%) of the participants in this group who continued their daily Tai Chi Qigong practice in groups informally after the completion of the three-month Tai Chi Qigong program. Some of the participants in this group eventually became friends. Notably, some participants came to the research center together during the six-month follow-up assessment, proving that their social network had been broadened after participating in the Tai Chi Qigong program and explaining why they experienced an improved level of satisfaction in perceived social support from friends at the study endpoint. Thus, social support needs time to build rapport and solid relationships among the participants. Moreover, social support may have influenced the participants' motivation to continue the Tai Chi Qigong exercise. Encouragement from their social support may have influenced the positive clinical and statistical significance for the TCQ group. The Tai Chi Qigong program is believed to create a supportive atmosphere for sharing interests and beliefs, which can then foster social support among the participants.

The exercise group and the control group did not show any significant changes in the perceived social support throughout the six-month study period. From the informal observation during the community activity classes, the participants did interact with one another; however, the interaction was limited to casual and superficial conversations. Expression in whatever form showing support and caring was rarely noticed. After the three-month class was completed, the participants did not have any continued interactions. Thus, although the community activities had created an environment for sharing of interest, such sharing was superficial, and the strength was not sufficient to facilitate the development of social support among the participants.

### 4.2. Limitations

First, the excessive attrition rate recorded in the control group could contribute to limitation, to a certain degree, in maintaining sample representativeness. The high attrition may have affected the validity of study results. Future studies should plan attractive placebo activities and arrange a Tai Chi Qigong wait-list plan in the control groups to retain participants and minimize attrition. Second, owing to the small number of female participants enrolled in this study, gender differences on the Tai Chi Qigong effects could not be examined. Future studies should recruit more female subjects, if available, so that gender differences on the therapeutic effects of Tai Chi Qigong can be evaluated.

### 4.3. Implications for Clinical Practice

The study provided new evidence that Tai Chi Qigong could be beneficial for long-term health promotion in COPD patients. In addition, Tai Chi Qigong is a safe and feasible intervention that causes no harm to the patients. Integrating Tai Chi Qigong into patients' daily activities provides a good opportunity for them to actively and independently participate in activities. Given that Tai Chi Qigong is a low-technology and gentle exercise, it is particularly appropriate and favorable for people with COPD as they are usually diagnosed at their later years of life and their physical functions have deteriorated because of normal aging process and disease progression. Tai Chi Qigong can work in conjunction with therapeutic treatment regimens and act as an alternative choice of exercise for health promotion and health maintenance in COPD patients.

## 5. Conclusions

The HRQL of individuals with COPD is affected by deteriorated physical functions, and their need for social support is increased. The three-month Tai Chi Qigong program indicates that Tai Chi Qigong is effective in psychosocial improvement among COPD patients. Continued self-practicing Tai Chi Qigong exercise sustains the beneficial effects for a longer period, significantly enhances HRQL, and increases self-perceived social support from friends. Its effectiveness, appropriateness, and feasibility for COPD patients make it a promising exercise option for this particular population.

## Figures and Tables

**Table 1:** Demographic and baseline data of subjects by group allocation [11]*.

	TCQ (*n* = 70)	Exercise (*n* = 69)	Control (*n* = 67)	*P* value
Age (Year), mean (SD)	71.7 (8.2)	73.6 (7.5)	73.6 (7.4)	0.24
Gender				0.02*
Male (%)	69 (99)	61 (88)	58 (87)	
Female (%)	1 (1)	8 (12)	9 (13)	
Years of COPD, mean (SD)	10.3 (9.3)	10.6 (8.8)	12.4 (10.6)	0.32
Marital status				0.51
Married (%)	64 (91.4)	60 (87.0)	54 (80.6)	
Single (%)	1 (1.4)	0 (0.0)	2 (3.0)	
Separated (%)	1 (1.4)	1 (1.4)	2 (3.0)	
Widowed (%)	4 (5.7)	8 (11.6)	9 (13.4)	
Live with				0.30
Alone (%)	4 (5.7)	4 (5.8)	8 (11.9)	
Family (%)	66 (94.3)	65 (94.2)	59 (88.1)	
Education level				0.38
Illiteracy (%)	9 (12.9)	10 (14.5)	14 (20.9)	
Primary (%)	41 (58.6)	47 (68.1)	37 (55.2)	
Secondary (%)	18 (25.7)	9 (13.0)	15 (22.4)	
Tertiary or above (%)	2 (2.9)	3 (4.3)	1 (1.5)	
Religious beliefs				0.33
Yes (%)	28 (40.0)	30 (43.5)	26 (38.8)	
No (%)	42 (60.0)	39 (56.5)	41 (61.2)	
Smoking status				0.81
Second hand smoker (%)	1 (1.4)	2 (2.9)	3 (4.5)	
Never smoke (%)	2 (2.9)	4 (5.8)	3 (4.5)	
Ex-smoker (%)	55 (78.6)	47 (68.1)	46 (68.7)	
Current smoker (%)	12 (17.1)	16 (23.2)	15 (22.4)	
Stage of COPD				0.50
Mild (%)	7 (10)	13 (19)	12 (18)	
Moderate (%)	31 (44)	26 (38)	29 (43)	
Severe (%)	32 (46)	30 (43)	26 (39)	
Coexisting diseases				
Heart disease (%)	9 (13)	10 (15)	6 (9)	0.58
HT (%)	28 (40)	36 (52)	28 (42)	0.21
DM (%)	7 (10)	6 (9)	7 (10)	0.95
No co-existing disease (%)	16 (23)	16 (23)	20 (30)	0.13
SGRQ-HKC, mean (SD)	42.7 (15.1)	37.0 (16.6)	39.4 (16.2)	0.11
Total MSPSS-C, mean (SD)	50.3 (18.1)	50.2 (17.1)	46.6 (16.4)	0.38

*Refer to reference [11].

HT: hypertension; DM: diabetes mellitus; SGRQ: St George's respiratory questionnaire; MSPSS: multidimensional scale of perceived social support; *P < 0.05.

**Table 2:** Comparison of SGRQ at baseline, 6th week, 3rd month, and 6th month.

	TCQ (*n* = 70)		Exercise (*n* = 69)		Control (*n* = 67)		Time *group *P* value	Partial eta squared
	Mean (SD)	Change from last measurement M (%)	Mean (SD)	Change from last measurement M (%)	Mean (SD)	Change from last measurement M (%)		
*Symptoms *							**<**0.001*	0.038
Baseline (T1)	45.75 (18.54)		38.64 (19.44)		37.89 (19.39)			
6th week (T2)	45.50 (17.85)	−0.25 (−0.55)	36.38 (20.24)	−2.26 (−5.85)	42.86 (18.96)	4.97 (13.12)		
3rd month (T3)	42.18 (19.03)	−3.32 (−7.30)	37.44 (18.17)	1.06 (2.91)	42.37 (20.07)	−0.49 (−1.14)		
6th month (T4)	41.11 (21.09)	−1.07 (−2.54)	40.90 (19.79)	3.46 (9.24)	43.13 (19.72)	0.76 (1.79)		

*Activity *							0.026*	0.023
Baseline (T1)	56.05 (19.82)		47.03 (21.65)		51.69 (21.24)			
6th week (T2)	52.43 (20.63)	−3.62 (−6.46)	52.43 (20.32)	5.40 (11.48)	53.42 (20.10)	1.73 (3.35)		
3rd month (T3)	54.43 (19.50)	2.00 (3.81)	53.51 (22.11)	1.08 (2.06)	54.10 (18.22)	0.68 (1.27)		
6th month (T4)	53.52 (22.27)	−0.91 (−1.67)	53.05 (20.45)	−0.46 (−0.86)	56.17 (18.56)	2.07 (3.83)		

*Impact *							0.030*	0.023
Baseline (T1)	34.11 (16.72)		30.67 (18.79)		32.77 (18.53)			
6th week (T2)	33.46 (17.10)	−0.65 (−1.91)	32.70 (18.56)	2.03 (6.62)	34.20 (16.59)	1.43 (4.36)		
3rd month (T3)	34.41 (17.34)	0.95 (2.84)	33.79 (17.85)	1.09 (3.33)	37.63 (16.72)	3.43 (10.03)		
6th month (T4)	32.49 (18.29)	−1.92 (−5.58)	35.27 (18.67)	1.48 (4.38)	37.48 (16.81)	−0.15 (−0.40)		

*Total SGRQ *							0.002*	0.034
Baseline (T1)	42.69 (15.13)		36.97 (16.56)		39.37 (16.18)			
6th week (T2)	41.22 (15.78)	−1.47 (−3.44)	39.31 (15.66)	2.34 (6.33)	41.48 (15.39)	2.11 (5.36)		
3rd month (T3)	41.77 (15.18)	0.55 (1.33)	40.39 (16.10)	1.08 (2.75)	43.41 (14.77)	1.93 (4.64)		
6th month (T4)	40.29 (16.94)	−1.48 (−3.54)	41.60 (15.74)	1.21 (3.00)	44.09 (15.01)	0.68 (1.57)		

SGRQ: St George's respiratory questionnaire.

Score ranges from 0 to 100, where “0” indicates the best health and “100” indicates the worst health.

*P < 0.05 for test of repeated measures ANCOVA.

**Table 3:** Comparison of MSPSS at baseline, 6th week, 3rd month, and 6th month.

	TCQ (*n* = 70)		Exercise (*n* = 69)		Control (*n* = 67)		Time *group *P* value	Partial eta squared
	Mean (SD)	Change from last measurement M (%)	Mean (SD)	Change from last measurement M (%)	Mean (SD)	Change from last measurement M (%)		
*Family *							0.602	
Baseline (T1)	21.50 (6.24)		22.55 (5.77)		21.39 (6.86)			
6th week (T2)	22.07 (6.48)	0.57 (2.65)	23.58 (5.42)	1.03 (4.57)	22.30 (5.23)	0.91 (4.25)		
3rd month (T3)	23.11 (5.98)	1.04 (4.71)	23.45 (5.02)	−0.13 (−0.55)	23.00 (5.41)	0.70 (3.14)		
6th month (T4)	23.39 (5.50)	0.28 (1.21)	23.19 (5.41)	−0.26 (−1.11)	22.57 (5.76)	−0.43 (−1.87)		

*Friend *							0.044*	0.023
Baseline (T1)	13.93 (7.87)		13.01 (7.51)		11.54 (7.72)			
6th week (T2)	13.31 (7.39)	−0.62 (−4.45)	13.28 (6.77)	0.27 (2.08)	13.85 (7.52)	2.31 (20.02)		
3rd month (T3)	14.63 (7.74)	1.32 (9.92)	12.87 (6.59)	−0.41 (−3.09)	13.70 (7.98)	−0.15 (−1.08)		
6th month (T4)	16.41 (7.70)	1.78 (12.17)	13.25 (6.93)	0.38 (2.95)	13.40 (7.37)	−0.03 (−2.19)		

*Significant other *							0.101	
Baseline (T1)	15.66 (7.96)		14.97 (7.68)		14.22 (7.84)			
6th week (T2)	14.87 (7.88)	−0.79 (−5.04)	15.99 (7.60)	1.02 (6.81)	15.22 (7.61)	1.00 (7.03)		
3rd month (T3)	16.20 (7.88)	1.33 (8.94)	15.19 (7.16)	−0.08 (−5.00)	15.60 (7.93)	0.38 (2.50)		
6th month (T4)	18.26 (6.84)	2.06 (12.72)	15.67 (7.53)	0.48 (3.16)	15.40 (7.31)	−0.02 (−1.28)		

*Total MSPSS *							0.056	
Baseline (T1)	51.09 (18.75)		50.54 (16.71)		47.18 (17.16)			
6th week (T2)	50.33 (18.12)	−0.76 (1.49)	52.84 (16.21)	2.30 (4.55)	51.37 (17.14)	4.19 (8.88)		
3rd month (T3)	53.94 (18.54)	3.61 (7.17)	51.51 (15.02)	−1.33 (−2.52)	52.30 (17.68)	0.93 (1.81)		
6th month (T4)	58.06 (17.04)	4.12 (7.64)	52.09 (16.63)		51.37 (16.73)	−0.93 (−1.78)		

MSPSS: multidimensional scale of perceived social support.

The total score ranges from 12 to 84, with higher scores indicating higher levels of perceived support.

*P < 0.05 for test of repeated measures ANCOVA.

## Abbreviation List

ANCOVA: Analysis of covariance
ANOVA: Analysis of variance
COPD: Chronic obstructive pulmonary disease
HQOL: Health related quality of life
MCID: Minimum clinically important difference
MSPSS: Multidimensional scale of perceived social support
SGRQ: St George's respiratory questionnaire
TCQ: Tai chi Qigong.
